# Evaluation of the Psychometric Properties of the Musculoskeletal Health Questionnaire (MSK-HQ) in a Population of Professional Basketball Players: A Cross-Sectional Study

**DOI:** 10.3390/medicina60040664

**Published:** 2024-04-19

**Authors:** Giovanni Galeoto, Kennet Matteo Viglianisi, Anna Berardi, Giovanni Sellitto, Ilaria Ruotolo, Rachele Simeon, Alessandra Carlizza

**Affiliations:** 1 Department Human Neurosciences, Sapienza University of Rome, 00185 Rome, Italy; anna.berardi@uniroma1.it (A.B.); giovanni.sellitto@uniroma1.it (G.S.); ilaria.ruotolo@uniroma1.it (I.R.); rachele.simeon@uniroma1.it (R.S.); 2 IRCSS Neuromed, Via Atinense, 18, 86077 Pozzilli, Italy; 3 UniCamillus, International Medical University in Rome, 00131 Rome, Italy; kv685@students.unicamillus.org (K.M.V.); alessandra.carlizza@unicamillus.org (A.C.)

**Keywords:** musculoskeletal injury, quality of life, internal consistency, Cronbach’s alpha

## Abstract

*Background and Objective:* Musculoskeletal disorders affect a large portion of the population worldwide. The musculoskeletal health questionnaire is a helpful tool for assessing the health state of patients with these disorders. The primary goal of this study is to evaluate the psychometric properties of the MSK_HQ-IT in a population of professional basketball players. The secondary aim is to assess the prevalence of musculoskeletal disorders. *Material and Methods:* The study was performed from September to October 2022. The questionnaire was completed using an online or paper form, to which personal data were collected. Data were collected by submitting a translated version of the musculoskeletal health questionnaire directly to professional athletes. *Results:* A total of 63 basketball players were recruited. Regarding functional limitations, the body parts mentioned by the sample were the left ankle (23.8%) and the right ankle (17.5%), followed by the lumbar column and right hip (15.9%). Regarding pain, the data are more homogeneous, with a distribution in line with functional limitations. A peak of pain was perceived in the left foot, with a mean score of 4. Cronbach’s alpha showed a value of 0.85. *Conclusions*: The musculoskeletal health questionnaire shows promising results in evaluating the health state of a population of professional athletes. Further studies are needed to enlarge the sample and possibly open it to more categories of professional sports.

## 1. Introduction

Basketball is a popular game practiced all over the world. A recent systematic review analyzed more than 12,000 basketball injuries from 11 included studies. The results showed that there were more injuries in the lower limbs (63.7% of the injuries), regardless of gender (male, 65.2%, female, 68.4%) or level (professionals, 64.7%, master 74.5%, and children and adolescents 62.5%).

According to the specific anatomical region, the largest proportion of injuries occurred in the ankle (2832 injuries, 21.9%), followed by the knee (2305 injuries, 17.8%). Most authors point to the ankle as the most common injury site; however, some authors report that the knee is the most affected region [1].

Regarding upper limb injuries, injuries to the hands, fingers, and wrists (1133, 8.7%) predominated over shoulder, arm, and forearm injuries (585, 4.5%) [2].

The unique demands of professional basketball place players at increased risk of sustaining specific orthopedic injuries [3]. Playing basketball requires a broad range of quick movements, including sprinting, cutting, pivoting, and jumping on a hardwood surface. A player performs an average of 1000 movements during a game, resulting in a change of action every 2 s, and 10% of playing time is spent in high-intensity activity [4].

A recent cross-sectional study of German athletes in different sports highlighted that 77% declared low back pain, followed by neck pain (63%) and thoracic spine pain (46%). A subsequent cross-sectional study of 1114 German athletes reported an 89% prevalence of low back pain [5]. A Finnish study, carried specifically on basketball players, highlighted that 46% of the participants in the study had low back pain [6].

Numerous studies have investigated the epidemiology of injuries in basketball players. No studies evaluate the quality of life in individuals with musculoskeletal disorders and professional basketball players.

Numerous scales investigate the evaluation of the generic quality of life, but none are specific for evaluating the quality of life associated with musculoskeletal disorders.

A Patient-Reported Outcome (PRO) is a health outcome directly reported by the patient, unlike other outcomes reported by health professionals (e.g., physician-reported outcome or nurse-reported outcome) [7].

The MSK-HQ is a short questionnaire that allows people with musculoskeletal conditions (such as arthritis or back pain) to report their symptoms and quality of life in a standardized way. It was developed jointly by the Arthritis Research UK Primary Care Sciences Research Centre at Keele University and the University of Oxford, co-produced with active participation and feedback from people with arthritis and musculoskeletal conditions, clinicians, and academics. The MSK-HQ enables patients and their therapists to monitor overall musculoskeletal health, rating its progress and response to treatment. Moreover, the questionnaire allows particular aspects of musculoskeletal health to be addressed, ensuring a holistic approach to patient needs, but it is also possible to consider individual components of the score, such as sleep quality or mood. Simply using the MSK-HQ may support people to report a wider range of their symptoms to their clinical team than can be measured by a simple clinical screening. The MSK-HQ, therefore, has the potential to become in musculoskeletal health what “blood pressure” is in cardiovascular health: an essential measure of musculoskeletal health that can be used throughout health systems for the benefit of people with musculoskeletal conditions. The measurement properties of the MSK-HQ have been proven in various musculoskeletal disorder patient samples [8]. In a study about the Danish version, the MSK-HQ seems to discriminate well between unchanged and improved patients across two cohorts. A key vision of the MSK-HQ was to produce a single broad health-status measure which was more sensitive to change than generic health tools. In both cohorts, the effect sizes of the MSK-HQ were considerably larger than those of the EQ-5D-5L, which indicates the superiority of the MSK-HQ, whereas for the ability of the MSK-HQ to discriminate between improved and unchanged patients (i.e., responsiveness) at 12 weeks, superiority was only observed with respect to the Danish cohort.

The questionnaire has 14 questions, which provide a score ranging from 0 to 56. There is also a question which will ask respondents to indicate, in the previous week, how many days they have engaged in physical activity that has raised their heart rate for 30 min or more (0 to 7 days) [9].

This scale (MSK-HQ-IT) has been validated in Italian on a healthy population and has shown excellent psychometric properties [10].

The MSK-HQ scale has never been used to evaluate a population of athletes and, furthermore, the scores on the scale have never been correlated with information regarding joint limitations and pain [11]. Every sporting practice is characterized by specific functional overload of all those joints most used during the motor gesture. For this reason, it is important to have validated tools capable of evaluating the specific impact that joint limitations have on the quality of life of athletes.

The primary goal of this study is to evaluate the psychometric properties of the MSK_HQ-IT in a population of professional basketball players. The secondary aim is to evaluate the prevalence of musculoskeletal disorders through a questionnaire to investigate limitations and joint pain of the various body segments.

## 2. Materials and Methods

The study was performed in accordance to the ethical standards of the 1964 Declaration of Helsinki and its later amendments.

### 2.1. Participants and Procedure

The scientific literature related to validation studies reports sample size recommendations ranging from 2 to 20 subjects per item [12,13]. A systematic review analyzed 114 validation studies and highlighted that the mean subject to item ratio was 28, with a minimum of 1 and a maximum of 527 [14]. Furthermore, Shoukri et al. [15] reports that “However, in many cases, values of the reliability coefficient under the null and alternative hypotheses may be difficult to specify. Under such circumstances, one can safely recommend only two or three replications per subject”. According to the “Consensus-based standards for the selection of health measurement instruments” (COSMIN) guidelines and consistent with previous studies of the MSK-HQ [16], a minimum sample size of 30 subjects was considered adequate for this study. The participants in this study are professional basketball players who play in the first three leagues of the Italian basketball federation, played in recent years, or are without a current contract but are practicing and training while they wait for a contract. The choice to take only professionals from the first 3 leagues is strictly connected to the fact that the workload in those leagues is basically the same, with some differences based on coaches’ choices. The study was performed from September to October 2022.

The MSK-HQ together with a questionnaire for collecting demographic data was administered online using the Google Forms application. All the material was self-administered, so to avoid bias in the validation of the instrument, the questionnaire was explained before compilation.

Eligible individuals were informed about the methods and objective of the study; specifically, they were informed that data would be provided without any identifier or group of identifiers which would allow the attribution of private information to an individual. People interested in participating signed an informed consent form [17,18].

### 2.2. Instruments

Data were gathered by submitting a questionnaire to a population of professional athletes.

This questionnaire is composed of four parts.

The first part is composed of the informed consent form of the study, in which we gather a few personal data from the participants, and the second part is composed of a personal data sheet. The third part is composed of a questionnaire that evaluates the musculoskeletal disorder, in particular, the functional limitation and/or pain. If pain is present, participants are asked to give a score of 0 to 10, where 0 indicates no pain, and 10 unbearable pain. The body parts investigated are the cervical, thoracic, and lumbar region of the spine; other parts considered are the shoulders, elbows, wrists, and hands for the upper body, and the hips, knees, ankles, and feet for the lower body.

The fourth part comprises the musculoskeletal health questionnaire (MSK-HQ). This part analyses the symptoms of joints and muscles, such as rigidity and pain. Those symptoms can be referred to the activities of daily life like dressing up, with a total of 14 questions, plus a question that estimates the level of physical activity. The score goes from 4 to 0, where 4 means that the patient does not experience pain, 3 means little pain, 2 means moderate pain, 1 means intense pain, and 0 is high-intensity pain [10].

### 2.3. Statistical Analysis

To conduct the statistical analysis, the standards of the COSMIN checklist were followed [19]. The internal consistency of the MSK-HQ was analyzed using Cronbach’s alpha. A fair, good, and excellent degree of internal consistency were considered with values of Cronbach’s alpha of 0.7, 0.8, and 0.9, respectively [20]. In order to assess the cross-cultural validity, correlations with the prevalence of musculoskeletal injury were measured. Statistical significance was set at *p* values < 0.05, and the IBM^®^ SPSS^®^ tool (Chicago, IL, USA) was used for the statistical analysis.

## 3. Results

### 3.1. Population

The included participants to this study were 63 basketball players; the mean age was 26.13 years old (Standard Deviation 4.7), and all players were male. The complete demographic characteristics of the sample are shown in Table 1.

### 3.2. Cross-Cultural Analysis

The cross-cultural analysis of functional limitations and pain are shown in Table 2.

#### 3.2.1. Functional Limitations

The participants showed no functional limitation only for the two hands and the left elbow. The parts that are more affected and predictable are the two ankles with a percentage of 23.8 for the left and 17.5 for the right. The two ankles are followed by the lumbar column and right hip, with 15.9% of participants answering the functional limitation question positively. The left hip has a limitation of 12.7%. We found a difference between the two knees that can be explained by the fact that most participants are right-handed, meaning they jump thousands of times more with the left knee for an athletic gesture learned in childhood (left knee 11.1% and right knee 7.9%). The cervical region does not show decreased functional limitations; just 3.2% of participants answered this question positively. The dorsal column was mentioned to have a limitation in 7.9% of participants, like both shoulders. Only 3.2% of the sample shows limitations in the right elbow.

The right wrist and both feet are mentioned by 4.8% of participants as parts with functional limitations. The left wrist is limited in functionality in 6.3% of the sample.

A total of 42.9% of the sample mentions having pain in the lumbar region, followed by the right ankle (38.1%) and the left ankle (33.3). The left knee is also where pain is felt, which is mentioned more than the right knee. The cervical column is mentioned by 19% of the sample as a part where the pain is felt. A total of 15.9% feel pain in the dorsal column. The right shoulder is painful in 20.6% of participants, and the left is painful in 17.5%. Both elbows are perceived as a source of pain (15.9% right and 12.7 left). Regarding wrists, the data show that of the whole sample, 15.9% feel pain on the right side, while 17.5% feel pain on the left side. Data in pain evaluation are more homogeneous, and so for hands, 19% of participants mentioned the right hand and 14.3% the left hand. A total of 27% of participants felt pain in the right hip, while 20.6 in the left. The difference between feet is relevant, and 30.2% of the sample feel pain in the left foot and 19% in the right.

#### 3.2.2. Pain

When giving a score for the pain, the average values range from 1.27 for the left elbow to 4 for the left foot. The average pain perceived in the cervical region is 1.75, the value for the dorsal region is 2, and that for the lumbar region is 2.67, one of the highest scores. The value for the right shoulder is 2.06, and that for the left shoulder is 2.36. The value for the left elbow is 1.27, while that for the right elbow is 1.54. Distally, the average pain is 1.38 for the right wrist and 1.86the left. The right-hand pain score is 1.73, while the left-hand score is 1.42. Proceeding caudally with the lower limbs, at the level of the hips on the right side, the pain is perceived at an average of 2.65, while at the left side just 1.94. The right knee scores high in pain with 2.84, and the left with 2.77. The value for the right ankle is 2.38 and for the left 2.30, while that for the right foot is 2.43.

### 3.3. Internal Consistency

The assessment of the reliability of the scale shows statistically significant data. Cronbach’s alpha showed a value of 0.85. This shows that the scale has high reliability. The alpha deleted analysis is presented in Table 3.

## 4. Discussion

This cross-sectional study falls under level 2b of scientific evidence which includes well-designed cohort studies. Level II studies produce evidence obtained from a well-designed cohort study or case–control analytic studies, preferably from more than one research center or group.

Although numerous studies on the health, musculoskeletal function, and injuries of basketball players have been published in the past decade [21,22,23,24], to date, no specific outcome measures have been validated to measure the quality of life related to musculoskeletal disorders in this population. This study represents the first study measuring the psychometric properties of an instrument measuring this construct for the professional basketball players. As we mentioned in the introduction, musculoskeletal disorders impact people’s lives worldwide with significant incidence.

The first aim of this study was to validate the MSK-HQ in Italian in a population of basketball players with a high risk of injuries.

Data were collected from 63 basketball players. Participants have an average age of 26.13 ± 4.7(18–37) and 18.3 ± 4.8 (8–35) hours of training. Therefore, the significant amount of time spent on high-impact training increased the possibility of injuries.

The internal consistency of the scale showed a Cronbach’s alpha value of 0.85; these data are statistically significant, and the alpha deleted analysis has shown that all items contribute to evaluating the internal consistency of the scale.

These data are consistent with the validation in Italian [10], which showed a value equal to 0.87 for a sample of healthy people. It is also consistent with the Arabic version, which showed a value of 0.88 [25]; the Hungarian version, which showed a value of 0.92 [8]; the Turkish version with a value of 0.91 [26]; and the Norwegian version with a value of 0.86 [27].

All the versions evaluated for comparison examine healthy populations, but none of these evaluate the quality of life of athletes. The secondary aim was to evaluate the prevalence of musculoskeletal disorders through a questionnaire to investigate the limitations and joint pain of the various body segments.

The highest prevalence was found for limitations of the two ankle joints, with a percentage of 23.8 for the left and 17.5 for the right. The two ankle joints are followed by the lumbar spine and the right hip, where 15.9% of participants responded positively to the question on functional limitation.

This result corresponds with the systematic review by de Carvalho Borges et al., which showed that the largest proportion of injuries occurred in the ankle (2832 injuries, 21.9%), followed by the knee (2305 injuries, 17.8%). Most authors point to the ankle as the most common site of injury; however, some authors report that the knee is the most affected region [28].

This study showed that the average pain perceived in the cervical region is 1.75/10; in the dorsal region, it is 2/20; and in the lumbar region, it is 2.67/10, one of the highest scores. These data are directly correlated to the functional limitation related to the lumbar area reported by 15.9% of the study participants. Most basketball professionals claim that lower back pain is present because the game involves frequent rotational movements at the lumbar level. Basketball players frequently suffer from low back and neck pain, which confers a high risk for spinal injuries, and lumbar spine injuries are common in basketball players [29].

A longitudinal study evaluated all injuries in the National Basketball Association (NBA) players over 17 years. The study highlighted that 10.2% of all injuries involved the lumbar spine, and 0.9% were due to lumbar disk degeneration [28].

Perfectly matched with the introduction of the study, those data show that lumbar pain, the leading cause of years lived with a disability due to musculoskeletal disorders, ranks high in pain and functional limitations. The same argument applies to the ankles, with lateral ankle sprain being the most common injury in professional basketball, accounting for 80,2% of all injuries [30].

Low back pain is a common problem in athletes. Clinicians must be able to identify athletes at high risk of low back injuries. Superficial heat and spinal manipulation therapy are the most strongly supported evidence-based therapies. Nonsteroidal anti-inflammatory medications and skeletal muscle relaxants have benefit in the initial management of low back pain; however, both have considerable side effects that must be considered. Athletes can return to play once they have recovered a full range of motion and have the strength to prevent further injury [31].

Riva et al. reported that improvements in proprioceptive control in single stance may be a key factor for an effective reduction in ankle sprains, knee sprains, and low back pain [32].

Further study needs to be undertaken regarding lower limbs. Data show that right-handed players have their left knee, ankle, and foot more affected by musculoskeletal disorders due to athletic gestures, with just the exception of pain perceived. Functional limitation and pain show great correlation and seem to be strictly connected in professional basketball players.

Perceived pain is relatively low in all body parts, probably because of the habit of practicing over it or because of the reluctance of athletes to talk openly about it. Therefore, studies that delve deeper into this argument can be developed.

This cross-sectional study has some limitations: the sample consisted only of male players; moreover, the sample size did not allow the evaluation of differences in scores with respect to the characteristics of the sample. It would be necessary to carry out further studies to evaluate the different populations of basketball players with respect to the level of athletic preparation and playing category. Furthermore, it would be interesting to carry out the same validation studies in other populations of athletes to have a shared tool and specific data on the quality of life in athletes. More studies with a larger group of athletes can be developed. There was great difficulty in finding the subjects for this study because of the reluctance of athletes to talk about their physical conditions. However, there is the prerequisite to enlarge the sample and open it to another category of athletes. The validation of the scale in a population of professional basketball players gave great results.

## 5. Conclusions

The availability of guidelines recommending the use of valid and reliable assessment tools and the more uniform reporting of outcome measures in studies on different athletic populations would allow comparisons across studies and enable the pooling of data from different studies for evidence synthesis. However, both research and clinical practice lack a consensus of a primary outcome measure. The MSK-HQ showed great reliability in evaluating the musculoskeletal disorders and how they affect quality of life in different populations and different countries. In conclusion, it may be recommendable for the clinic and for studies concerning the epidemiology, prevention, and treatment of these disorders.

## Figures and Tables

**Table 1:** Sample characteristics.

	Sample N°63
Age Mean ± DS (RANGE)	26.13 ± 4.7 (18–37)
Height (in meters) Mean ± DS (RANGE)	1.96 ± 0.08 (1.80–2.15)
Weight (in Kg) Mean ± DS (RANGE)	94.13 ± 9.58 (77–118)
Hours per week of sports activity Mean ± DS (RANGE)	18.3 ± 4.8 (8–35)

**Table 2:** Assessment of musculoskeletal disorders.

	Functional Limitation		Pain		
	No	Yes	No	Yes	Means ± DS
	N° (%)				
Cervical spine	61 (96.8)	2 (3.2)	51 (81)	12 (19)	1.75 ± 1.06
Backbone	58 (92.1)	5 (7.9)	53 (84.1)	10 (15.9)	2.00 ± 1.472
Lumbar spine	53 (84.1)	10 (15.9)	36 (57.1)	27 (42.9)	2.67 ± 1.348
Right shoulder	58 (92.1)	5 (7.9)	50 (79.4)	13 (20.6)	2.06 ± 1.289
Left shoulder	58 (92.1)	5 (7.9)	52 (82.5)	11 (17.5)	2.36 ± 1.646
Right elbow	61 (96.8)	2 (3.2)	53 (84.1)	10 (15.9)	1.54 ± 0.967
Left elbow	63 (100)		55 (87.3)	8 (12.7)	1.27 ± 0.647
Right wrist	60 (95.2)	3 (4.8)	53 (84.1)	10 (15.9)	1.38 ± 0.650
Left wrist	59 (93.7)	4 (6.3)	52 (82.5)	11 (17.5)	1.86 ± 1.099
Hand Dx	63 (100)		51 (81)	12 (19)	1.73 ± 0.884
Hand Sx	63 (100)		54 (85.7)	9 (14.3)	1.42 ± 0.669
Anca Dx	53 (84.1)	10 (15.9)	46 (73)	17 (27)	2.65 ± 1.631
Anca Sx	55 (87.3)	8 (12.7)	50 (79.4)	13 (20.6)	1.94 ± 1.237
Right knee	58 (92.1)	5 (7.9)	47 (74.6)	16 (25.4)	2.84 ± 1.893
Left knee	56 (88.9)	7 (11.1)	43 (68.3)	20 (31.7)	2.77 ± 1.232
Right ankle	52 (82.5)	11 (17.5)	39 (61.9)	24 (38.1)	2.38 ± 1.525
Left ankle	48 (76.2)	15 (23.8)	42 (66.7)	21 (33.3)	2.30 ± 1.460
Right foot	60 (95.2)	3 (4.8)	51 (81)	12 (19)	2.43 ± 1.950
Left foot	60 (95.2)	3 (4.8)	44 (69.8)	19 (30.2)	4.00 ± 3.256

**Table 3:** Alpha deleted analysis.

Item	Medium Scale If the Item Is Deleted	Scale Variance If the Element Is Deleted	Correct Element-to-Total Correlation	Quadratic Multiple Correlation	Cronbach’s Alpha If the Item Is Deleted
1	41.68	29,252	0.603	0.481	0.841
2	41.10	28,636	0.548	0.506	0.844
3	41.00	30,000	0.501	0.445	0.846
4	40.67	30,000	0.609	0.605	0.842
5	41.49	28,609	0.613	0.525	0.839
6	40.98	27,661	0.783	0.720	0.829
7	40.68	31,220	0.459	0.576	0.849
8	40.67	31,452	0.351	0.361	0.854
9	40.95	31,756	0.255	0.363	0.860
10	41.97	33,967	−0.006	0.237	0.868
11	41.13	27,242	0.675	0.518	0.835
12	41.56	29,122	0.503	0.596	0.847
13	41.51	30,706	0.383	0.505	0.853
14	41.57	27,894	0.692	0.603	0.834

## Data Availability

Data supporting this study’s findings are available from the corresponding author upon reasonable request.
